# Protecting Vulnerable Road Users from Injury

**DOI:** 10.1371/journal.pmed.1000228

**Published:** 2010-03-30

**Authors:** Aymery Constant, Emmanuel Lagarde

**Affiliations:** Equipe Avenir prévention et prise en charge des traumatismes, Centre de recherche INSERM U897 “Epidémiologie et Biostatistiques,” Université Victor Segalen Bordeaux 2, France

## Abstract

Aymery Constant and Emmanuel Lagarde discuss policies to protect pedestrians, and pedal and motor cyclists, from injury.

## Introduction

In its latest global assessment of road safety, the World Health Organization (WHO) reminded us that half of the 1.2 million fatalities occurring each year on the world's roads concern vulnerable road users (VRUs), with children and elderly being overrepresented among victims [1]. “Vulnerable road user” is a term applied to those most at risk in traffic, i.e. those unprotected by an outside shield [2]. Pedestrians, pedal cyclists, and motor cyclists are accordingly considered as vulnerable since they benefit from little or no external protective devices that would absorb energy in a collision. They constitute with almost no exception the weak party in a road traffic crash. The aim of this article is to provide an overview of the global health problem posed by VRU injuries. Priorities regarding prevention initiatives for VRUs and barriers to effective policies are discussed in the context of both industrialised and developing countries.

## The Burden of VRU Injuries

With a high and increasing proportion of VRUs, developing countries are facing a major public health challenge regarding VRU injuries [1],[3]. For example, motorised two-wheelers account for more than 70% of road traffic deaths in Thailand and 44% in Colombo, Sri Lanka. Similarly, pedestrians account for 42% of all road traffic deaths in Delhi, India, and 38% in Colombo, Sri Lanka. With continuing urbanisation and motorisation, VRU injuries and deaths represent a serious threat to their development and jeopardise the pursuit of equity in health [4],[5]. Preoccupying trends in VRU injuries are also observed in industrialised countries where the modal part of VRUs has recently increased in urban areas, due to environmental, economic, and traffic congestion issues. In the United States, the latest data show a decrease in fatalities for all road users except for motorcyclists and cyclists [6]. In France, where traffic law enforcement has dramatically increased since 2002, the proportion of car users among road fatalities decreased by 16% from 1997 to 2007, while those of VRUs increased by 25% over the same period [7]. According to the European Transport Safety Council [8], the death risk per 100 million person kilometres travelled is 13.8 for motorised two-wheelers, 6.4 for pedestrians, and 5.4 for bicyclists in Europe. This is to be compared with a death risk of 0.7 for car users and 0.07 for bus and coach passengers. The severity of VRU injuries is also higher than those of four-wheelers. For all these reasons, the awareness of the challenge posed by VRU injuries is now moving to the top of the agenda of international aid organisations [9]. Based on the WHO World Report on Road Traffic Injury Prevention, a recent United Nations resolution encourages Member States to increase road safety efforts with special attention towards VRUs [10].

## The Problem of Poor Data Collection

The weakness of data collection by police forces in the aftermath of a crash involving a VRU has been documented using comparisons with hospital records. Pedestrians' and cyclists' nonfatal crashes are heavily underestimated. In the European Union, traffic injury statistics in 2004 recorded only 12% of cyclists' injuries [11]. Even in the case of severe injuries, the police very seldom record cyclist crashes that do not involve other vehicles. These crashes are often wrongly considered as non-traffic crashes as the bicycle is perceived more as a vehicle for leisure or sport than for transport. This view is all the more inappropriate as the bicycle is increasingly considered as a valid alternative to other transportation means with health and environmental benefits.

## General Traffic Policies that Would Benefit All VRUs

Ways to address VRU road safety concern all road users and are expected to lead to significant improvement in overall road safety, including VRUs. Three main areas have been extensively investigated with available evidence-based results: speed, alcohol, and visibility/conspicuity.

Speed plays a key role in road safety as it increases both crash risk and crash severity. This is all the more true for VRUs who cannot count on the car body as protection and deceleration buffer. Consequently, speed mitigation policies clearly benefit all VRUs. When struck by a car at 45 km/h, less than 50% of pedestrians or cyclists survive. At 30 km/h, more than 90% survive [12]. Efforts to reduce speeding include speed limit setting and enforcement and traffic-calming engineering measures (speed bumps, chicanes, roundabouts) [13]. Lowering the speed limit in dense areas is probably the most effective and affordable intervention to stem traffic crashes in both high- and low-income countries [14],[15]. Law enforcement, however, is costly and subject to corruption, a phenomenon not only observed in developing countries [16]. Systematic review of controlled before/after studies showed that traffic-calming engineering measures may have the potential to reduce road traffic deaths and injuries, but their effects in developing countries still need to be assessed further [17].

Drinking and driving is the other main cause of traffic injuries, and setting and enforcing blood alcohol limits is therefore essential. However, its mitigated success led to consideration of a more restrictive solution. According to a systematic review, alcohol ignition interlocks, which prevent drivers from starting the engine if their blood alcohol level is over the legal limit, appear to be effective when the device is installed in the vehicle of potential offenders [18].

Visibility (sufficient range of unobstructed vision) and conspicuity (being clearly discernible) are fundamental in preventing traffic crashes. One of the basic driver errors responsible for collisions is the late detection of other road users [19]. In the United States, 67% of all fatal vehicle-pedestrian collisions occur at night [20]. Systematic analyses of the U.S. Fatality Analysis Reporting System database indicate that pedestrian fatalities increase as illumination decreases even when other factors are held constant [21],[22]. Evidence from systematic reviews indicates that street lighting may prevent road traffic crashes, injuries, and fatalities for all road users, especially VRUs [13],[23]. But further well-designed studies are still needed to determine the effectiveness of street lighting in developing countries [23]. Conspicuity aids (lamps, flashing lights, retroreflective materials) also have the potential to improve detection and recognition and merit further development to gain public acceptance [24], especially from cyclists and pedestrians travelling in rural areas without road lights and in urban areas with poor lighting. Preliminary findings from a Web-based survey in a large cohort of cyclists indicate that low cyclist conspicuity may increase the risk of crash-related injury [25]. A systematic review shows, however, that the actual impact of conspicuity aids on cyclist safety remains to be measured [26]. Finally, because underdevelopment is most often associated with poor street lighting, conspicuity aids are of particular interest in developing countries where cyclists and pedestrians are often not properly visible [27].

## Countermeasures That Are Specific to Motor and Moped Cyclists

As far as motorised two-wheelers are concerned, the most effective protection that can be offered is the helmet. Evidence from a systematic review shows that it reduces the risk of fatal injuries by 42% [28]. The rate of helmet use is high in developed countries but often very low elsewhere [29]–[32], due to inadequate awareness, regulation, and enforcement [33]. New protective devices such as jacket air-bags are being developed but are still subject to reliability issues. Anti-lock Braking Systems and Combined Braking Systems (front and rear brakes are applied by a single means of control) proved helpful in preventing a significant number of falls [34], but their generalisation faces cost barriers. Effective roadway improvements to decrease the risk to riders of motorised two-wheelers include skid-resistant road marking, road maintenance (minor defects can be a safety hazard for cyclists and motorcyclists), and efforts towards a more forgiving roadside, such as the replacement of safety barriers with motorcycle-friendly safety barriers.

## Countermeasures That Are Specific to Pedal Cyclists and Pedestrians

Modifications of the built environment can substantially reduce the risk of severe injuries among pedestrians and cyclists by separating them from motorised traffic. According to reviews of evidence-based engineering interventions [13],[35], sidewalks and refuge islands are of interest to protect pedestrians from collisions with motor vehicles, while bicycle facilities (e.g. on-road bike routes, off-road bike paths) are associated with the lowest risk for cyclists. The high cost of modifying the built environment requires that bicycle and pedestrian facilities be installed on a limited basis in locations where collisions are most likely to occur—for instance, in inner-city centres, between contiguous neighbourhoods, and along major arterial streets [36],[37]. The use of simple artwork such as on-road bicycle lanes might be of interest in countries with few financial resources or in less prioritised areas.

When it comes to cyclists, a systematic review shows that helmet use results on average in a 70% reduction in the risk of head injuries [38], but its use is mandatory in a limited number of countries, and encouraged in some. There is controversy over the relevance of mandatory use, which has been hypothesized to be a deterrent to bicycle use or to cause helmeted cyclists to behave less carefully [39]. More research is needed in this area to assess how the local context may influence the impact of helmet promotion and of coercive rules. Another frequent cause of fatal injuries is the situation when a truck makes a turn without noticing the cyclist. Both truck and cycle drivers should be made aware of this common hazard. Truck enhanced mirror systems and side underrun protection are also effective measures.

The prevention of pedestrians' injuries is more complex, as walking in the street is often considered a common life activity carrying no particular hazard. Those with immature or impaired perception and cognitive skill (children, elderly, alcohol-intoxicated pedestrians) are particularly vulnerable. New four-wheeler vehicles are increasingly designed to be less injurious to pedestrians and other VRUs. However, if designing safer car fronts is important, we will have to wait for several years to record a significant impact on morbidity and mortality statistics, especially in developing countries, where vehicles are older. Vehicle onboard advanced sensing systems are currently being developed to track road users and assist in preventing or reducing pedestrian injuries. However, while technology-based strategies (including the design of safer car fronts) might have a significant impact on VRU fatalities in industrialised countries, their costs will limit their use in developing and middle-income countries [40], where they are the most needed.

Awareness prevention campaigns have remained relatively scarce among cyclists and pedestrians, who are consequently sometimes unaware of road hazards or consider that crash avoidance is up to motorists only. Favouring the weakest road users is legitimate, as they are both more vulnerable and less hazardous to other road users. But this needs to be accompanied by enhanced assessment and prevention of VRU risk behaviours. For instance, the public should be made fully aware that drinking is a risk not only among users of motor vehicles but also among pedestrians [41],[42] and cyclists [43],[44]. Because they share the same pathways as motorists, cyclists and sometimes also pedestrians should be expected to obey the same restrictive rules concerning risky behaviours, including alcohol intoxication. Changing behaviours and attitudes might also be pivotal in reducing road casualties in developing and middle-income countries, where large populations of cyclists, pedestrians, and moped cyclists interact with motorised transport in unforgiving infrastructures [3]. An extensive literature has investigated cross-cultural differences in attitudes toward road safety, showing that compliance with traffic rules, risk perception, and safe behaviour vary widely according to cultural factors, social norms, and habits [45],[46]. Internalisation of social norms requires understanding as to why they are of value or why they make sense [47],[48]. Accordingly, evidence-based interventions might be effective if they consider shifting road users' beliefs from contextual and cultural schemes that might favour unsafe behaviours (e.g. fatalistic theory of injury as an act of God [49], beliefs that health issues cannot be prevented and use of ineffective prevention measures [50], patriarchal notions of masculinity that admire toughness and risk-taking [51]) to attitudes favouring safer practices (e.g. knowledge of injury severity sustained by VRUs, increased awareness of road risk, increased understanding of other road user behaviours). Evidence from a systematic review indicates that pedestrian safety education can change observed road crossing behaviour [52]. But whether this reduces the risk of pedestrian injury in road traffic crashes is still unknown. Education programs targeting pedestrians might, however, be of interest, especially if culturally adapted and accessible to large audiences in low- or middle-income countries. Because in many pedestrian crashes the driver reportedly does not see the pedestrian before the collision, they should include a focus on the dangers of interacting with traffic and on the use of conspicuity aids, especially at night [53].

## Conclusion

VRU traffic injuries are the greatest challenge of today's worldwide road safety. We still lack data to assess the actual extent of the burden and much remains to be done to investigate all potential solutions. However, as is often the case in road safety, only a multipronged approach will be successful, combining passive and active devices with regulations, enforcement, and awareness campaigns. Developing countries could learn much from the experience of the industrialised countries regarding the framework for injury control. However, injury prevention interventions targeting VRUs in low- and middle-income countries have to overcome additional challenges related to cost, feasibility, sustainability, and a higher level of traffic mix with an already high and increasing proportion of VRUs. A pivotal point is that VRU behaviours should not be balanced against other users' behaviours. VRUs have a high traffic injury risk and are therefore not exempted from obeying traffic rules. When traffic separation is not possible, other users need to learn how to safely share their road space with more vulnerable users with different behaviours, speed, situational awareness, and conspicuity.

**Key Policy Recommendations to Protect Vulnerable Road Users**

- Encourage cooperation between police forces, emergency departments, and public health agencies in order to improve data collection on VRU injuries
- Raise all road users' awareness of the particular behaviours and risks of VRUs.
- Develop prevention campaigns specifically oriented towards VRUs in order to:
  - Increase awareness of road hazards
  - Promote injury prevention (helmet use, conspicuity aids, etc.)
  - Increase the perception of risk related to alcohol use when cycling or walking.
- In dense urban areas, traffic engineering countermeasures should include:
  - Measures to reduce speed of motorised traffic
  - Measures to improve street lightning when visibility is inadequate
  - Measures to segregate motorised and nonmotorised road users, especially in locations where collisions are most likely to occur.

## Abbreviations

VRU: vulnerable road user
